# Majestic tigers: personality structure in the great Amur cat

**DOI:** 10.1098/rsos.220957

**Published:** 2023-04-05

**Authors:** Rosalind Arden, Abdel Abdellaoui, Qian Li, Yao Zheng, Dengfeng Wang, Yanjie Su

**Affiliations:** ^1^ Centre for the Philosophy of the Natural and Social Sciences, London School of Economics, London, UK; ^2^ Department of Psychiatry, Amsterdam UMC, University of Amsterdam, Amsterdam, The Netherlands; ^3^ School of Psychological and Cognitive Sciences and Beijing Key Laboratory of Behaviour and Mental Health, Peking University, Beijing, People's Republic of China; ^4^ Department of Psychology, University of Alberta, Edmonton, Alberta, Canada

**Keywords:** tiger, animal personality, animal behaviour, cats, great cats, conservation

## Abstract

We explore individual differences in tiger personality. We first asked—is there evidence of personality dimensions (analogous to the Big Five in human personality research) in the Amur tiger? We then asked, are any discoverable personality dimensions associated with measured outcomes, including group status, health and mating frequency? 152 of our participating tigers live in the world's largest semi-wild tiger sanctuary in North Eastern China. Our second sample of 96 tigers also lives in a sanctuary. Having two samples allowed us to assess the replicability of the personality dimensions or factors reported in our first sample. We found that two factors (explaining 21% and 17% of the variance among items) which we call, for descriptive ease, Majesty and Steadiness, provide the best fit to the data. Tigers that score higher on Majesty are healthier, eat more live prey, have higher group status (among other tigers as assessed by human raters) and mate more often. We provide some ethological context to put flesh on the quantitative bones of our findings concerning these magnificent and charismatic animals.

## 1. Background

### 1.1. Animal personality

Anyone who spends much time with several mammals of the same species is likely to observe personality differences among individuals. Among mammals, human personality is the most studied and a lexical approach was developed to probe it (see for review [1]). We adopt this approach in exploring our focal species, the greatest cat on earth: the Siberian or Amur tiger, *Pantheris tigris altaica*.

All tigers are not the same. We cannot know how the character or temperament of one tiger strikes another, but we can assess how the personality of a tiger strikes human observers. Given enough observations and enough tigers, we can ask ‘does tiger personality reduce somewhat to a set of orthogonal dimensions—spectra along which individuals within a species have been found to vary in their behavioural responses and dispositions (see for example [2])?’ This was our first research question.

### 1.2. Siberian or Amur tiger

The year we wrote this paper, 2022, was the year of Tiger in the Chinese lunar calendar; an auspicious time as well as a scientifically critical time to learn about this animal because they are endangered [3–5]. This is partly because of their own magnificence—tigers attract medicine hunters whose clients seemingly consider that the virility and charisma of the tiger are transmissible by consuming its body parts [6,7]. Threats to tigers also arise because they compete with people for territory. Poaching, logging and the growth of towns and cities have eroded the vast forests which sustain them [8,9]. Now, of all times, we should be alert to possible harms that may arise from the incautious use of wild animal parts (see for review [10]). Further, the Amur nuclear genome closely resembles that of the South China tiger [11], considered extinct in the wild. This provides another scientific motivation for protecting the Amur tiger.

Amur tigers are the largest cats (an adult male can reach around 3 m in length and weigh up to 206 kg [12]). They have the biggest range of any cat—in males up to 2000 km^2^ [12]. These tigers can live to at least 14 years in the wild and have lived up to 35 years in captivity [12].

Amur tigers are obligate carnivores. They prey on wild boar, red deer, bears, bucks and birds [13]. It takes great skill to survive in low prey density territory, which is reflected in cubs being moderately altricial. Cubs stay with their mother for 2–3 years, and a father has been observed provisioning cubs [14], which is rare among cats. Tigers are sensitive to changes in their environment—such as new or altered objects since last passing along, say, a hunting track. As evidence of this, notes Sooyong Park—a renowned tiger specialist who locked-down in a small, ice-covered bunker for six months to observe and film Siberian tigers—despite his camouflage efforts, a mother tiger spotted a well-hidden camera lens. ‘She let out an indescribable, blood-curdling growl, and the attack began. She caught the zoom cable that was dragged out with the lens when it fell off and yanked at it … [she] clawed off the camouflage shrubs … ’ [15, p. 133]. This hard-won observation is relevant to tiger personality since traits such as vigilance, wariness, boldness, in addition to cognitive abilities, almost certainly influence survival and reproduction in a demanding resource-scarce environment. Fitness consequences of personality traits have been assessed empirically; these vary by ecology, species and sex. We expect that the offspring of more vigilant mothers, as in the example above, have a survival advantage [16]. Tigers have the largest cranial volume among cats [17], which is consistent with the conjecture that Amur tiger survival and reproduction is cognitively demanding.

Tragically, there are more tigers living in Texas (estimate around 5000 [18]) than the roughly 500 Amur tigers living in the wild [19]. Not that this indicates something amiss with Texas, but there is much amiss with such large-brained and wide-ranging animals being in held in captivity. We hope that scholarship on these magnificent animals will encourage support for their welfare and conservation in the wild and in protected reserves (see for example [20]).

### 1.3. The lexical approach to probing personality

Human personality research arises from a lexical viewpoint [1]. The premise of this approach is that because people think and talk about themselves and others, human personality should be captured in our language by adjectives that describe us (such as kind, avaricious, mendacious or reliable). The next step is to test whether data reduction techniques can reveal an underlying (not directly observable) pattern or structure to these words when applied to individuals. In human personality research, a leading model supports the existence of five factors (openness to experience, conscientiousness, extraversion, agreeableness and stability), which capture many of the differences among people [21]. The work on tigers reported here follows the same general strategy (discussed in [22], see ch. 2).

We asked the following questions: (i) is there a discernible structure of personality in Amur tigers? (ii) is any personality in tigers linked to other outcomes such as physical characteristics, or social status? (iii) is there evidence of sex differences in tigers? To explore these questions, we assessed data from two tiger populations.

## 2. Methods

### 2.1. Sample One

All the animals in this report are semi-captive. Animals in Sample One live in the largest protected reserve in China, the Harbin Siberian Tiger Park, in North Eastern China, which extends 1.44 km^2^. The study comprises 152 tigers (85 males and 67 female tigers, age range 1–16 years). In the park, tigers are fed in the open ground. All tigers had lived for more than six months in the park.

### 2.2. Sample Two

Ninety-six tigers (52 males, 44 females, age range 2–18 years) live in Hengdaohezi Siberian Tiger Park, Hailin city, Heilongjiang province, 272 km from Harbin. The densely forested park borders a mountain and extends 0.14 km^2^. In this park, tigers are separated by age-group and fed in the open ground. Each tiger had lived in the park for at least six months.

### 2.3. Measures

A tiger personality questionnaire containing a list of 70 words (items) considered suitable to describe tiger personality was given to each rater. Each word was defined in writing. Please see the electronic supplementary material for a step-by-step description of the scale-development. For the second sample three words were dropped (because of low eigenvalues), so the questionnaire administered to Sample Two contained 67 words.

### 2.4. Raters

The raters of the first, larger, sample of 152 tigers comprised 26 people who were either feeders or veterinarians. Each rater had worked with the tigers for at least six months and could identify every individual tiger. The raters were instructed in the use of a seven-point Likert scale, the meanings and nuance of any adjectives were clarified, and behaviours associated with various adjectives were discussed between raters and researchers so that the raters shared a consensus view of what they were being asked to do. The rating questionnaires were completed over a two-week period. The same process was followed for Sample Two.

The 27 raters of the second, smaller, sample of 96 tigers were also feeders or veterinarians, with at least one year of experience working at the park. All raters could identify each tiger and were familiar with the tigers’ physical characteristics and behaviours. The rating questionnaires were completed over a one-week period.

### 2.5. Study design

Within each sample, all raters were invited to rate all tigers. In fact, 475 questionnaires were returned from the larger sample, and 340 questionnaires were returned from the second smaller sample, providing a high item-to-rater ratio. For Sample One, there were between 1 and 12 raters per tiger, with an average of 3.1 raters per tiger. For Sample Two, the number of raters ranged from 1 to 8 raters per tiger, with an average of 3.6 raters per tiger.

### 2.6. Analytical strategy

#### 2.6.1. Rater effects

We first quantified the rater effects, on each sample, using the intraclass correlation coefficient (ICC) to assess the extent to which different people assessing the same tiger agreed on their evaluations. For the ICC, we used a one-way random effects model estimating the reliability for the average of *k* raters using *ICC* from the *psych* package in R 4.1.2 [23,24].

Next, using *lm* from the *stat* package in R 4.1.2, we ran regressions on dummy variables created for each rater in order to estimate the explained variance by all raters and to obtain residuals without rater effects. We created a set of item responses with and without rater effects for each dataset (samples one and two) for follow-up analyses.

#### 2.6.2. Principal components analysis

Using *princomp* from the *stat* package in R 4.1.2, we conducted a principal components analysis (PCA) on Sample One to get an indication of the magnitude of any dimensions of variation that emerged from the 70 items.

#### 2.6.3. Exploratory factor analysis

Guided by the number of potential underlying factors suggested by the PCA and previous work on other cats [25], we conducted an exploratory factor analysis (EFA) on Sample One using *factanal* from the *stat* package in R 4.1.2. This approach uses maximum likelihood (ML), which assumes that the observed variables come from a mixture of several Gaussian distributions, that is, the latent variables each come from a unique Gaussian distribution that has some noise. As in most animal behaviour research, we did not assess fit indices due to their requirements of large sample sizes and high sensitivity to normality assumptions [26,27]. Instead, we look for the most parsimonious solution while relying on the interpretability of the factors and their meaningfulness. We performed two EFAs: one on the dataset with rater effects and one on the dataset without rater effects.

#### 2.6.4. Factor analysis with Procrustes Rotation

Following exploratory factor analyses, we conducted on both samples a factor analysis (FA) with Procrustes Rotation, guided by its utility as a confirmatory tool [28] using *factanal* from the *stat* package in R 4.1.2. We then tested for congruence between the two samples as our measure of repeatability across the two samples, both with rater effects left in and rater effects regressed out in R 4.1.2 as described in [28].

#### 2.6.5. Associations between personality factors and some evolutionarily relevant outcomes

Taking the best fitting model forward from the factor analyses with Procrustes Rotation, we explored correlations among the factors and measured outcomes: age, length, weight, health, preying on live animals, food intake, mating frequency, breeding, rank among other tigers as assessed by human observers, and whether a tiger was raised by his or her mother (rather than human-fed). We explored these correlations separately for each sample. Correlations were computed using *rcorr* from the *Hmisc* package in R 4.1.2.

#### 2.6.6. Sex differences in evolutionarily relevant outcomes

Lastly, we examined sex differences in the factors and each of the above-measured outcomes, separately within each sample of tigers, using *t.test* from the *stats* package in R 4.1.2.

## 3. Results

### 3.1. Rater effects

**Figure 1.**
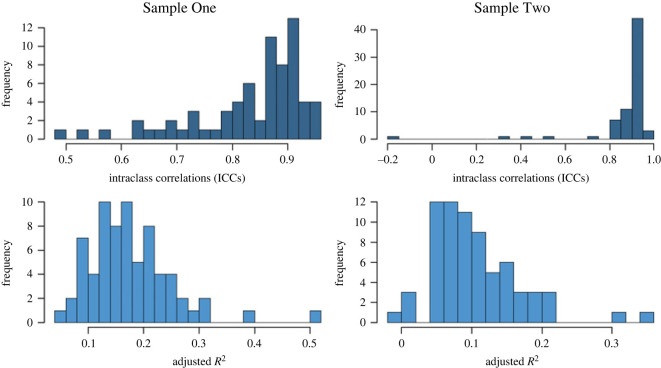
Distributions of item-level rater effects for both samples. The upper two plots show the distributions of the intraclass correlations (ICC) for the 70 items from the one-way random effects models with raters as random effects. The average ICC is 0.83 for Sample One and 0.87 for Sample Two, indicating an overall good reliability of the average scores of raters. The lower plots show the distributions of the explained variance for the 70 items of the raters when coded as dummies in a regression analysis. The average *R*^2^ is 0.18 for Sample One and 0.10 for Sample Two.

We computed the ICCs in a one-way random effects model, with raters as random effects. This model was devised for datasets where the same set of raters report on all subjects; it estimates the reliability of the average score of all raters for a single item [23]. On average, the 70 items had an average ICC of 0.83 (ranging from 0.49 to 0.95) for Sample One, and an average ICC of 0.87 for Sample Two (ranging from −0.16 to 0.96). These values indicate a good reliability of the average scores of raters [23], but there was variation in the ICCs between items (figure 1), which is why we created two sets of items for each sample: one taking the average item score between raters for each tiger, and one where we regressed out the rater effects before taking the average item score between raters for each tiger. We regressed out the rater effects using dummy variables for the raters, of which the distributions of the explained variance (*R*^2^) are shown in figure 1, with an average *R*^2^ of 0.18 for Sample One and 0.10 for Sample Two.

### 3.2. Principal components analysis

**Figure 2.**
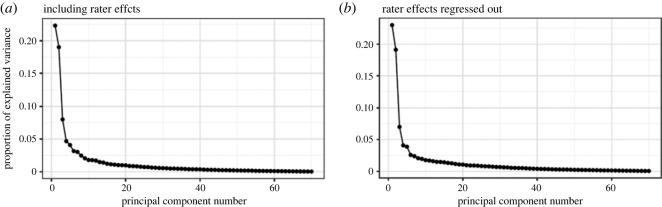
Scree plot of the PCAs conducted on Sample One. The two scree plots show the explained variance for each principal component (PC) for the PCA done on Sample One with rater effects included (*a*) and rater effects regressed out (*b*).

The scree plot of the PCA conducted on Sample One indicated the existence of two major factors explaining greater than 15% of the variance each, a third major factor explaining greater than 5%, and two additional smaller factors explaining approximately 4% each. These results were similar whether raters were left in or regressed out (figure 2).

### 3.3. Exploratory factor analysis

Based on the PCA results, we ran exploratory factor analyses on Sample One with two-, three- and five-factor models. The most parsimonious options contained two or three factors, as the number of items with factor loadings greater than 0.4 dropped substantially for the fourth and fifth factors (table 1). As in most animal behaviour research, fit indices were not considered due to their requirements of large sample sizes and high sensitivity to normality assumptions [1,26,27].

**Table 1.** The number of items with factor loadings stronger than 0.4 for the two-, three- and five-factor models for Sample One, including rater effects (left) and with rater effects regressed out (right).

	including rater effects			rater effects regressed out		
	2 factors	3 factors	5 factors	2 factors	3 factors	5 factors
Factor 1	34	34	32	33	32	32
Factor 2	25	24	21	31	24	29
Factor 3	—	19	16	—	18	8
Factor 4	—	—	7	—	—	6
Factor 5	—	—	3	—	—	3

The three-factor solution resulted in factors explaining approximately 20%, approximately 15% and approximately 10% of the variation (electronic supplementary material, figure S1). Only the first two factors showed much overlap of items with factor loadings greater than 0.4 between the EFA including rater effects and the EFA with rater effects regressed out (32 and 11 items overlap, respectively, with factor loadings correlating 0.98 and 0.99; electronic supplementary material, figure S2). The factors of the two-factor model explained approximately 20% and approximately 18% of the variation (electronic supplementary material, figure S3). Both factors showed a large overlap of items, with factor loadings greater than 0.4 between the EFA including rater effects and the EFA with rater effects regressed out (31 and 23 items overlap, respectively, with factor loadings correlating 0.98 and 0.99; electronic supplementary material, figure S4). These results suggest that removing the rater effects matters only for the third factor, but not for the first two major factors.

### 3.4. Factor analysis with Procrustes Rotation

To evaluate the replicability of the two- and three-factor structures, we conducted an EFA in the replication sample, using targeted, i.e. Procrustes, rotations to assess the factor similarity with the first sample. When exploratory factor analyses in independent datasets show similar factor structures, it is considered strong evidence of replicability. This approach has been shown to produce more reliable results than confirmatory factor analyses (CFAs) in evaluating the replicability of factor structures in human personality data [28]. The quantitative index used to evaluate factor similarity is the congruence coefficient used in [28], where a value higher than 0.9 is considered a matching factor, and higher than 0.8 considered a fair similarity [28,29].

**Figure 3.**
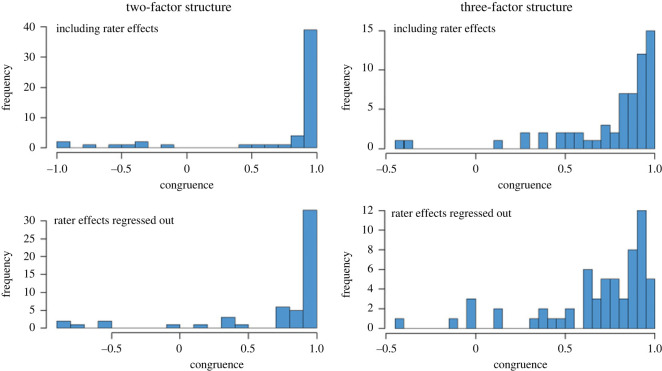
Distributions of the item-level factor loading congruence of the EFAs between Sample One and Sample Two. The left panels show the results of the two-factor structure, of which the items with the rater effects left in showed a higher overall congruence (0.81) than the items with rater effects regressed out (0.77). The right panels show the results of the three-factor structure, of which the items with the rater effects left in showed a higher overall congruence (0.78) than the items with rater effects regressed out (0.71).

**Figure 4.**
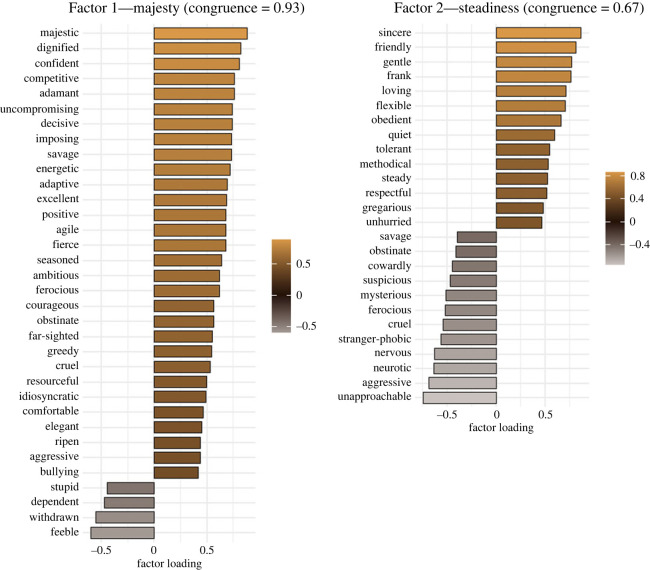
The factor loadings of the two-factor model with Procrustes Rotation including rater effects. This figure shows the solution with the highest overall congruence between the two samples which will be used in the follow-up analyses.

We included items with a factor loading of greater than 0.4 in the initial EFA. The new EFAs were conducted in Sample One and Sample Two with Varimax rotations. These replication analyses were conducted with rater effects left in and with rater effects regressed out. For the three-factor model, the average factor congruence was generally higher with rater effects left in (per factor congruence: Factor 1: 0.89, Factor 2: 0.76, Factor 3: 0.64) than with rater effects regressed out (per factor congruence: Factor 1: 0.80; Factor 2: 0.67; Factor 3: 0.67), although both overall congruence coefficients were lower than 0.8 (overall congruences with rater effects left in and with rater effects regressed out were 0.78 and 0.71, respectively; figure 3 and electronic supplementary material, figure S5). The two-factor structure showed higher congruence coefficients than the three-factor structure, and again, the items with the rater effects left in (Factor 1: 0.93, Factor 2: 0.67) showed a higher overall congruence than items with rater effects regressed out (Factor 1: 0.67, Factor 2: 0.87). The best results overall were obtained for the two-factor structure with rater effects left in, with an overall congruence of 0.81, with the first factor showing the highest congruence of all (0.93; figures 3 and 4 and electronic supplementary material, figure S6). As expected, the correlation between these two factors is small; 0.17 (*p* = 0.03) in the first dataset and 0.16 (*p* = 0.12) in the second dataset. These are the two factors that will be included in all subsequent analyses.

Since a factor number is an impoverished interpretive label, we refer to Factor 1 as Majesty, and Factor 2 as Steadiness guided by the adjectives comprising the items in the two factors (provided in electronic supplementary material)*.* We draw attention to the richness of the Chinese language in personality assessment (data collection and ratings were conducted by Chinese native speakers).

### 3.5. Associations between personality factors and some evolutionarily relevant outcomes

Table 2 below shows correlations between the two factors, Majesty, and Steadiness, in each sample. Tigers scoring higher in Majesty were healthier, preyed more on live animals, ate more, mated more often, bred more often, and were regarded by their human raters as having higher group status among tigers. Since some of the tigers had been fed by humans as nurselings, we could determine that being mother-reared was associated (not necessarily causally) with being more Majestic. There was broad comparability across the two samples. Only one sex difference showed in these correlational data. In the second and smaller dataset, the link between Majesty and being reared by his or her mother was stronger in males (*p* = 0.002 two-tailed). This could be sampling variance since it was not found in the first and larger sample.

**Table 2.** Pearson correlations between factors and baseline characteristics (partial *R* = corrected for sex and age).

	factor	age	length	weight	health	preying	food intake	mating freq.	breeding	rank in the herd	raised by mother
Sample One											
all (*N* = 152)	Majesty	*0.21	0.12	*0.24	***0.36	***0.38	***0.38	***0.33	***0.28	***0.47	***0.39
	Steadiness	−0.12	−0.02	0.05	*0.21	*0.23	0.10	0.04	−0.05	*0.18	*0.18
partial *R* (*N* = 152)	Majesty	*n.a.*	0.02	0.14	***0.42	***0.37	***0.32	**0.27	*0.24	***0.43	***0.35
	Steadiness	*n.a.*	0.04	0.13	*0.20	**0.28	*0.17	*0.20	0.06	*0.24	*0.22
males (*N* = 85)	Majesty	*0.27	0.12	0.18	*0.22	*0.24	*0.24	**0.34	*0.33	***0.39	**0.35
	Steadiness	−0.05	0.08	0.10	0.11	0.14	0.16	0.17	0.00	0.16	*0.25
females (*N* = 67)	Majesty	0.14	−0.04	0.14	***0.48	***0.47	***0.42	*0.32	*0.27	***0.48	**0.39
	Steadiness	−0.19	−0.05	0.14	*0.32	***0.47	0.16	−0.09	−0.11	*0.27	0.16
Sample Two											
all (*N* = 96)	Majesty	*−0.26	*0.27	0.18	**0.31	0.04	*0.28	*0.29	*0.26	**0.34	*0.29
	Steadiness	−0.01	0.03	0.05	0.15	−0.05	0.07	0.05	0.15	0.01	0.10
partial *R* (*N* = 96)	Majesty	n.a.	***0.42	*0.22	***0.38	−0.02	***0.30	*0.22	**0.27	***0.35	*0.23
	Steadiness	n.a.	0.14	*0.20	*0.18	−0.05	0.10	0.06	*0.18	0.06	*0.12*
males (*N* = 43)	Majesty	*−0.43	−0.22	0.08	0.03	0.14	0.05	0.32	*0.50	**0.49	***0.63
	Steadiness	−0.02	−0.06	0.07	0.11	−0.12	0.09	0.21	*0.38	0.01	0.24
females (*N* = 41)	Majesty	−0.29	*0.37	0.25	*0.31	0.12	*0.44	*0.39	0.12	0.31	0.21
	Steadiness	0.00	0.12	*0.35	0.29	0.04	0.09	−0.14	−0.07	0.04	−0.04

*p < 0.05; **p < 0.0025 (Bonferroni within sex); ***p < 0.0006 (Bonferroni within all).

### 3.6. Sex differences observed among the Amur tigers

As expected, males were larger and heavier than females in both samples (electronic supplementary material, figure S7). Males preyed on live animals and ate more in the larger sample but not in the second sample. Males were more Majestic (*p* = 0.01, two-tailed) in the larger sample but not in the second sample.

## 4. Discussion

In this study of Amur tiger personality, we found evidence of two factors which together explain 38% of the variance in the questionnaire scores. These factors were largely replicable across two independent samples.

Why concern ourselves with probing tiger personality structure? In other animals individual differences in personality or behaviour have been associated with health [2] and breeding status [3]. As well as the intrinsic pleasure of learning more about the world we live in, research on animal personality can augment our capacity to manage and conserve wildlife more effectively.

We face obstacles in linking this work to similar studies since this is the first psychometric study on personality in wild-living Amur tigers we know of. An analysis of domestic cat personality recommends using the human five-factor model labels for dimensions (to avoid each researcher inventing their own labels which is a barrier to cross-study comparison) [30], yet we found the items and factor names for human personality a poor match to tiger personality. An interesting cross-site study of zoo-living Amur tigers found three personality factors called by the researchers ‘anxious, quiet and sociable’ but the sample size of 19 Amur tigers is small [31]. Our inventory of items was purpose-built for tigers in the large samples we were able to obtain. In naming our factors Majesty and Steadiness, we chose labels that are semantically coherent, even in translation. ‘Majesty’ includes ‘dignified, imposing, far-sighted’ and ‘ambitious’ to pick a few of the word items. ‘Steadiness’ includes ‘sincere, methodical, tolerant’ and, our favourite, ‘frank’. Both factors comprise items that, according to human values, are broadly positive; the undesirable trait items such as (stupid, dependent, aggressive and neurotic) load negatively on these two factors.

Majesty was linked with better health and higher status in both datasets. What in humans are desirable personality traits may co-occur with valuable outcomes in tigers. We wish to avoid going beyond our data in interpreting these results; it is possible that natural and sexual selection have forged a tendency for ‘good things to go together’ in tiger personality, but that is simply a suggestion. We can only assess tiger status from a step removed—our measure is the human raters’ assessment of the tigers’ ranking. But this measure was highly convergent among raters who had a great deal of observation time as well as expertise in tiger behaviour. Status is an evolutionarily significant trait among tigers. Male tiger fitness is enhanced by the acquisition and management of large territories; by limiting other males’ mating access to females within the territory, and by remaining healthy. ‘Status’ likely comprises a constellation of traits on which individuals assess one another. Status is relevant to tiger decision-making about when and whether to fight, or with whom to mate—choices that are highly consequential.

The Steadiness factor, which has items that could be interpreted as relating to a neuroticism-like dimension, had a lower congruence (0.67). One possible limitation that may have contributed to the lower congruence coefficient is sample characteristics. The replication sample may have had different characteristics that could have affected the factor structure. In only the second sample, for example, the tigers were separated by age-group and lived on a smaller territory. Another limitation could be measurement error, due to, for example, raters that differed in how long they knew the tigers (the raters in Sample One worked for the tigers for at least six months, in Sample Two for at least a year).

The only sex difference that manifested in both samples was the propensity for males to be heavier. We consider the other outcomes associated with being a male tiger (eating more, eating more live prey and being higher in the factor we call Majesty) only as possible true sex effects which may instead arise from sampling variance since they did not emerge clearly in both datasets.

We suspect that if one could study a large sample of tigers in the wild, the results could have been slightly different. It is unlikely that wild animals would express exactly the same behavioural repertoire as either a managed wild population, or a zoo population of the same species. We make no prediction about the size or direction of any such differences. Given the challenge in observing even a single wild tiger, our protected sanctuary-living tigers are the only currently practical possibility for collecting a psychometric dataset. The factors Majesty and Steadiness are linked with biological outcomes; this increases the likelihood that they are meaningful.

We hope this report will stimulate further work on Amur tiger personality. Managing land resources among competing species (in this case mostly humans and tigers) is a complex multivariate problem. This work shows that, like us, tigers are individuals. And that their temperaments are associated with ecologically relevant outcomes. There is much yet to learn about their individual and species-typical capacities. Let us hope enough tigers remain for future scholars to study, since we are short on immortal hands and eyes to frame replacements should we lose this species that burns so brightly in our imaginations and in life.

## Data accessibility

The data are provided in electronic supplementary material [32].

## References

[1] John OP, Angleitner A, Ostendorf F. 1988 The lexical approach to personality: a historical review of trait taxonomic research. Eur. J. Pers. **2**, 171-203. (10.1002/per.2410020302)

[2] Adams MJ et al. 2015 Personality structure and social style in macaques. J. Pers. Soc. Psychol. **109**, 338-353. (10.1037/pspp0000041) 26030054

[3] Luan X et al. 2011 Habitat evaluation of wild Amur tiger (*Panthera tigris altaica*) and conservation priority setting in north-eastern China. J. Environ. Manag. **92**, 31-42. (10.1016/j.jenvman.2010.08.001) 20828917

[4] World Wildlife Fund. Tigers [Internet]. World Wildlife. [cited 2022 Jan 24]. See https://www.worldwildlife.org/species/tiger.

[5] Journalist E species. 2022 The Status of Tigers in the Wild. Tigers in Crisis. See https://tigersincrisis.com/the-status-of-tigers/.

[6] Davis EO, Willemsen M, Dang V, O'Connor D, Glikman JA. 2020 An updated analysis of the consumption of tiger products in urban Vietnam. Glob. Ecol. Conserv. **22**, e00960. (10.1016/j.gecco.2020.e00960)

[7] Dinerstein E et al. 2007 The fate of wild tigers. Bioscience **57**, 508-514. (10.1641/B570608)

[8] Dou H, Yang H, Feng L, Mou P, Wang T, Ge J. 2016 Estimating the population size and genetic diversity of Amur tigers in Northeast China. PLoS ONE **11**, e0154254. (10.1371/journal.pone.0154254) 27100387 PMC4839643

[9] Tian Y, Wu J, Smith AT, Wang T, Kou X, Ge J. 2011 Population viability of the Siberian Tiger in a changing landscape: going, going and gone? Ecol. Modell. **222**, 3166-3180. (10.1016/j.ecolmodel.2011.06.003)

[10] Huang Q, Wang F, Yang H, Valitutto M, Songer M. 2021 Will the COVID-19 outbreak be a turning point for China's wildlife protection: new developments and challenges of wildlife conservation in China. Biol. Conserv. **254**, 108937. (10.1016/j.biocon.2020.108937) 33518771 PMC7833061

[11] Armstrong EE et al. 2021 Recent evolutionary history of tigers highlights contrasting roles of genetic drift and selection. Mol. Biol. Evol. **38**, 2366-2379. (10.1093/molbev/msab032) 33592092 PMC8136513

[12] Wildlife Conservation Society. 2021 Amur tiger ecology [Internet]. [cited 2022 Jan 24]. See https://russia.wcs.org/en-us/Wildlife/Amur-Tigers/Amur-Tiger-Ecology.aspx

[13] Yang H et al. 2018 Seasonal food habits and prey selection of Amur tigers and Amur leopards in Northeast China. Sci. Rep. **8**, 6930. (10.1038/s41598-018-25275-1) 29720702 PMC5931987

[14] Worrall S. 2015 How A Photographer Captured the Beauty of Siberian Tigers [Internet]. National Geographic.com. [cited 2022 Jan 24]. See https://www.nationalgeographic.com/adventure/article/151025-natural-history-siberian-tiger-siberia-poaching-russian-mafia-ngbooktalk

[15] Sooyong P. 2017 Great soul of siberia. London, UK: William Collins.

[16] Smith BR, Blumstein DT. 2008 Fitness consequences of personality: a meta-analysis. Behav. Ecol. **19**, 448-455. (10.1093/beheco/arm144)

[17] Yamaguchi N, Kitchener AC, Gilissen E, Macdonald DW. 2009 Brain size of the lion (*Panthera leo*) and the tiger (*P. tigris*): implications for intrageneric phylogeny, intraspecific differences and the effects of captivity. Biol. J. Linnean Soc. **98**, 85-93. (10.1111/j.1095-8312.2009.01249.x)

[18] Leigh H. 2020 5 Things Tiger King doesn't explain about captive tigers [Internet]. World Wildlife.org. [cited 2022 Jan 24]. See https://www.worldwildlife.org/stories/5-things-tiger-king-doesn-t-explain-about-captive-tigers

[19] Wildlife Conservation Society. 2021 Siberian tiger project [Internet]. See https://russia.wcs.org/en-us/projects/siberian-tiger-project.aspx

[20] Veasey JS. 2020 Can zoos ever be big enough for large wild animals? A review using an expert panel assessment of the psychological priorities of the Amur Tiger (*Panthera tigris altaica*) as a model species. Animals **10**, 1536. (10.3390/ani10091536) 32878205 PMC7552275

[21] McCrae RR, Costa Jr PT. 2008 The five-factor theory of personality. In Handbook of personality: theory and research, pp. 159-181, 3rd edn. New York, NY: The Guilford Press.

[22] Vonk J, Weiss A, Kuczaj SA. 2017 Personality in nonhuman animals. Springer International Publishing.

[23] Koo TK, Li MY. 2016 A guideline of selecting and reporting intraclass correlation coefficients for reliability research. J. Chiropr. Med. **15**, 155-163. (10.1016/j.jcm.2016.02.012) 27330520 PMC4913118

[24] Shrout PE, Fleiss JL. 1979 Intraclass correlations: uses in assessing rater reliability. Psychol. Bull. **86**, 420. (10.1037/0033-2909.86.2.420) 18839484

[25] Gartner MC, Powell DM, Weiss A. 2014 Personality structure in the domestic cat (*Felis silvestris catus*), Scottish wildcat (*Felis silvestris grampia*), clouded leopard (*Neofelis nebulosa*), snow leopard (*Panthera uncia*), and African lion (*Panthera leo*): a comparative study. J. Comp. Psychol. **128**, 414. (10.1037/a0037104) 25111629

[26] Budaev SV. 2010 Using principal components and factor analysis in animal behaviour research: caveats and guidelines. Ethology **116**, 472-480. (10.1111/j.1439-0310.2010.01758.x)

[27] Fabrigar LR, Wegener DT, MacCallum RC, Strahan EJ. 1999 Evaluating the use of exploratory factor analysis in psychological research. Psychol. Methods **4**, 272. (10.1037/1082-989X.4.3.272)

[28] McCrae RR, Zonderman AB, Costa Jr PT, Bond MH, Paunonen SV. 1996 Evaluating replicability of factors in the revised NEO personality inventory: confirmatory factor analysis versus procrustes rotation. J. Pers. Soc. Psychol. **70**, 552-566. (10.1037/0022-3514.70.3.552)

[29] Lovik A, Nassiri V, Verbeke G, Molenberghs G. 2020 A modified tucker's congruence coefficient for factor matching. Methodology **16**, 59-74. (10.5964/meth.2813)

[30] Litchfield CA, Quinton G, Tindle H, Chiera B, Kikillus KH, Roetman P. 2017 The ‘Feline Five’: an exploration of personality in pet cats (*Felis catus*). PLoS ONE **12**, e0183455. (10.1371/journal.pone.0183455) 28832622 PMC5568325

[31] Bullock N, James C, Williams E. 2021 Using keeper questionnaires to capture zoo-housed tiger (*Panthera tigris*) personality: considerations for animal management. J. Zool. Bot. Gard. **2**, 650-663.

[32] Arden R, Abdellaoui A, Li Q, Zheng Y, Wang D, Su Y. 2023 Majestic tigers: personality structure in the great Amur cat. Figshare. (10.6084/m9.figshare.c.6492826) PMC10073900 37035292

